# Associations of metabolic syndrome with minimal hepatic encephalopathy in patients with cirrhosis and portal hypertension: a retrospective cohort study

**DOI:** 10.3389/fnbeh.2026.1779609

**Published:** 2026-05-20

**Authors:** Xinyi Ding, Lingna Jing, Lei Li, Chao Zhao, Long Gao, Rong Wang, Chunjuan Zhao, Lijun Yang, Duiping Feng, Jinyu Li

**Affiliations:** 1 Academy of Medical Sciences, Shanxi Medical University, Taiyuan, Shanxi, China; 2 Department of Oncological and Vascular Intervention, First Hospital of Shanxi Medical University, Taiyuan, Shanxi, China; 3 Shanxi Provincial Clinical Research Center for Interventional Medicine, First Hospital of Shanxi Medical University, Taiyuan, Shanxi, China; 4 Department of Pharmacology, Shanxi Medical University, Taiyuan, Shanxi, China; 5 Department of Special Medicine, Shanxi Medical University, Taiyuan, Shanxi, China

**Keywords:** cirrhosis, diabetes, dose-response association, metabolic syndrome, minimal hepatic encephalopathy

## Abstract

**Background and purpose:**

Metabolic syndrome and minimal hepatic encephalopathy are both associated with impaired hepatic metabolic function. This study aimed to investigate their relationship in patients with cirrhosis and portal hypertension.

**Methods:**

This retrospective cohort study enrolled 127 patients. Minimal hepatic encephalopathy was diagnosed using the Psychometric Hepatic Encephalopathy Score. Restricted cubic spline and threshold analyses were used to assess dose-response relationships. Multivariable logistic and modified Poisson regression analyses were performed to identify independent predictors.

**Results:**

The prevalence of minimal hepatic encephalopathy was 17.3% (22/127). Patients with MHE had significantly higher fasting blood glucose (8.17 ± 4.36 vs. 5.33 ± 0.98 mmoL/L), and higher prevalence of metabolic syndrome and diabetes. Fasting blood glucose was an independent risk factor for MHE (*P* < 0.05), and diabetes showed a similar association in a separate model. A nonlinear relationship was observed between FBG and MHE risk (P for overall = 0.006, P for nonlinear = 0.078), with an exploratory inflection point at 8.36 mmol/L. Below this threshold, each 1 mmol/L increase in glucose corresponded to a 92.1% higher risk of MHE. Spearman correlation analysis showed that higher FBG levels were associated with poorer performance on DST and SDT subtests of PHES. Multivariate regression identified older age (OR = 1.103, *P* = 0.007), higher Child–Pugh grade (OR = 5.869, *P* = 0.008), and higher FBG (OR = 1.750, *P* = 0.007) as independent risk factors for MHE.

**Conclusion:**

Metabolic disorders, especially hyperglycemia is closely associated with the occurrence of MHE in cirrhotic patients. Blood glucose control and comprehensive metabolic management may help reduce the risk of MHE.

## 1 Introduction

Minimal hepatic encephalopathy (MHE) is a common complication of liver dysfunction (Gairing et al., 2023; Luo et al., 2023; Nardone et al., 2016; Rose et al., 2020), occurring in 20–80% of cirrhosis cases. It is associated with decreased quality of life and increased risk of hospitalization, and the risk of developing overt hepatic encephalopathy as well as increased mortality (Gairing et al., 2024a; Wang et al., 2017; Wong et al., 2014). The liver dysfunction is often accompanied by metabolic disorders of related lipids such as triglycerides (TG), high-density lipoproteins (HDL-C), and glucose metabolism disorders (Yunchong et al., 2024; Zichun et al., 2024). Studies have reported that about 80% of patients with cirrhosis suffer from glucose metabolism disorders, and blood lipid levels can also affect the prognosis of patients with cirrhosis, resulting in a series of complications (Zichun et al., 2024). This suggests that metabolic disorders resulting from liver dysfunction may be associated with the development of hepatic encephalopathy.

Metabolic syndrome (MetS) is not a disease but a cluster of multiple metabolic abnormalities related to liver metabolic disorders. It primarily encompasses hyperinsulinemia, impaired glucose tolerance, dyslipidemia, hypertension, and obesity, with insulin resistance being the core feature (Kassi et al., 2011; Khunti and Davies, 2005; Lemieux and Després, 2020). In recent years, MetS has become one of the major global public health issues and has received increasing attention (Alberti et al., 2005; Noubiap et al., 2025). Accumulating evidence suggests that diabetes is a key factor in the pathogenesis of hepatic encephalopathy (HE), involving hyperammonemia, intestinal dysbiosis, and systemic inflammation, and that patients with cirrhosis and diabetes are more likely to develop MHE than those with euglycemic cirrhosis (Ampuero et al., 2013; Coman et al., 2021; Elkrief et al., 2014). Annalisa Berzigotti found that obesity is a risk factor for the progression of compensated cirrhosis to decompensated cirrhosis (Berzigotti et al., 2011; Gu et al., 2023). Previous studies on the relationship between MetS and cognitive function have focused on the effects of single diseases such as diabetes and obesity on cognitive function. Diabetes and obesity are generally recognized as risk factors for cognitive impairment (Livingston et al., 2020; Strachan et al., 2011). However, research on the impact of MetS on cognitive impairment, especially on hepatic encephalopathy, is relatively scarce.

While individual MetS components have been implicated in HE pathogenesis, their synergistic effects remain unclear and identifying the predictors of HE is of great significance for constructing a more optimized screening process for high-risk patients. This study focuses on the intrinsic relationship between metabolic syndrome and its components with MHE, and evaluate the dose-response relationship.

## 2 Materials and methods

### 2.1 Patients

The data for this study originates from the Taiyuan Cirrhosis & Portal Hypertension Cohort Study (Li et al., 2023), which focuses on patients with cirrhosis and portal hypertension treated at the First Hospital of Shanxi Medical University from June 2022 to December 2024. Patient history, clinical features, underlying causes of cirrhosis, and prognostic information were collected from the medical records. Blood samples (for biochemical indicators such as FBG, blood lipids and PHES tests were both completed on the day of patient enrollment or within 24 h to ensure temporal consistency between biochemical assessment and psychometric evaluation.

Patients taking any psychotropic drugs within 3 months and organic neurologic pathologies (e.g., dementia or stroke) were excluded. A total of 127 patients were included in the study. All 17 patients with alcoholic cirrhosis had been abstinent for ≥ 6 months before enrollment, verified by medical history and family confirmation. Prior to enrolling the subjects, all participants gave written informed consent and the study was approved by the Research Ethics Committee of the First Hospital of Shanxi Medical University (K-K0050).

### 2.2 Metabolic syndrome

Data related to MetS were collected at the time of patient enrollment. According to the diagnostic criteria for MetS in the “Guidelines for the Prevention and Treatment of Type 2 Diabetes in China (2017 Edition)” (Metabolic Syndrome Research Collaboration Group of the Diabetes Branch of the Chinese Medical Association, 2004) formulated by the Chinese Diabetes Society (CDS) (meeting three or more of the following criteria): ➀ Overweight and/or obesity (body mass index ≥ 25 kg/m^2^); ➁ Fasting blood glucose (FBG) ≥ 6.1 mmoL/L and/or 2-h post-load blood glucose ≥ 7.8 mmoL/L, and/or those diagnosed with diabetes and receiving treatment; ➂ Systolic blood pressure (SBP) ≥ 140 and/or diastolic blood pressure (DBP) ≥ 90 mmHg, or those diagnosed with hypertension and receiving treatment; ➃ TC ≥ 1.7 mmoL/L and/or HDL-C < 0.9 mmoL/L (male), < 1.0 mmoL/L (female). The CDS criteria were chosen because they are specifically recommended for the Chinese population by the national diabetes society. Compared with Western criteria (e.g., NCEP-ATP III or IDF), the CDS criteria use a BMI cutoff of ≥ 25 kg/m^2^ to define overweight/obesity instead of waist circumference. This cutoff is more appropriate for Asian populations, as the World Health Organization recommends a lower BMI threshold for overweight ( ≥ 25 kg/m^2^) in Asians than in Caucasians ( ≥ 30 kg/m^2^). Using Western waist circumference criteria could underestimate metabolic risk in our cohort. Therefore, the CDS criteria provide a more accurate classification of MetS in this Chinese patient population.

### 2.3 MHE diagnosis

MHE was assessed using the PHES scale, an internationally recognized gold standard for diagnosing MHE (Riggio et al., 2011; Xu et al., 2025). The PHES scale comprises five subtests (Weissenborn et al., 2001): the Number Connection Test A (NCT-A), Number Connection Test B (NCT-B), Line Tracing Test (LTT), Serial Dotting Test (SDT), and Digit Symbol Test (DST). PHES scores have been standardized according to the age and education level of the Chinese population, referencing international guidelines and local validation studies. An abnormality in each subtest of PHES is defined as a value < −1, and a sum of all subtests < −4 indicates MHE (Ehrenbauer et al., 2023; Gairing et al., 2024b; Lauridsen et al., 2024). All tests were performed under the guidance of trained professionals to ensure accuracy and reliability.

### 2.4 Laboratory measurements and pre-analytical specifications

Blood samples were collected from all participants by standard venipuncture after an 8–12 h overnight fast. All specimens were obtained in the morning between 6:00 and 8:00 a.m. to minimize circadian variation. Serum was separated promptly by centrifugation at 3,500 rpm for 10 min within 2 h of collection and immediately analyzed using a fully automatic biochemical analyzer with commercially available kits. All laboratory procedures were performed in strict accordance with the standard operating protocols (SOPs) of the Clinical Laboratory of the First Hospital of Shanxi Medical University. Quality control samples were analyzed daily to ensure accuracy and precision of the test results.

### 2.5 Statistical analysis

Data were analyzed using SPSS statistics version 19.0 (IBM, Armonk, NY, United States) and R software version 4.3. Continuous variables are expressed as mean ± SD. Categorical variables are described by frequency (proportion). Categorical data and ordinal categorical variables are evaluated by chi-square tests for independence and trend, respectively. *T*-tests and Mann-Whitney U-tests are used to assess continuous standard and non-parametric data, respectively. After preliminary data processing, we used regression models to analyze whether MetS is associated with MHE. The RCS was used to evaluate the dose-response relationship between MetS and MHE risk. The threshold effect of MetS on MHE is further analyzed by a two-stage linear regression model. Modified Poisson regression analysis (Shan et al., 2023) was also performed to determine the adjusted relative risk (aRR) of MHE. Because the prevalence of MHE in our cohort was 17.3% (> 10%), logistic regression may overestimate the odds ratio; therefore, modified Poisson regression with robust error variance was used to directly estimate adjusted relative risks. In addition, to address potential collinearity among MetS components, variance inflation factors (VIFs) were calculated, with VIF > 5 indicating exclusion from multivariate models. Sensitivity analyses were performed by excluding patients with overt HE history or lactulose use. In all cases, a bilateral *P* < 0.05 is considered statistically significant.

The sample size was calculated for the primary outcome (association between fasting blood glucose and minimal hepatic encephalopathy) using the standard formula for two independent groups:


n=[(Zα/2+Zβ)×2(σ1+2σ2)2]/(μ1-μ2)2

where Z_α/2_ = 1.96 (two-sided α = 0.05), Z_β_ = 0.84 (power = 80%), μ1 and μ2 represent group means, and σ1 and σ2 represent standard deviations. Detailed results are shown in Supplementary Table S2.

## 3 Results

### 3.1 Baseline characteristics of the study population

A total of 127 patients were included in this study. MHE occurred in 22 (17.3%) patients. Patients with MHE had a higher mean age, more women, and higher Child-Pugh Class compared to those without MHE (all *P* < 0.05). They also had higher FBG (8.17 ± 4.36 mmoL/L vs. 5.33 ± 0.98 mmoL/L) and lower HDL-C (0.80 ± 0.25 mmoL/L vs. 0.96 ± 0.29 mmoL/L) levels, and patients with diabetes (50.0% vs. 21.9%) and MetS (40.9% vs. 14.3%) had a higher chance of developing MHE. There was no significant difference between MHE patients and without MHE patients in terms of other components of MetS, such as body mass index and blood pressure (all *P* > 0.05). The detailed baseline demographic and clinical characteristics of the study population are listed in Table 1.

**TABLE 1:** Demographics and clinical characteristics in this study (*n* = 127).

Variables	Total(*n* = 127)	MHE (*n* = 22)	No MHE(*n* = 105)	χ ^2^/*t*	*P*
**Age(years)**	56.48 ± 10.32	65.14 ± 8.52	54.67 ± 9.76	−4.669	**< 0.001**
**Gender(male)**	76 (59.8%)	7 (31.8%)	69 (65.7%)	8.696	**0.004**
**PHES scores**	−1.30 ± 3.20	−6.77 ± 1.57	−0.15 ± 2.06	14.240	**< 0.001**
BMI(kg/m^2^)	23.38 ± 3.77	22.81 ± 4.33	23.50 ± 3.65	0.780	0.437
Cirrhosis etiology				5.359	0.149
Hepatitis	54 (42.5%)	5 (22.7%)	49 (46.7%)		
Alcoholic	17 (13.4%)	4 (18.2%)	13 (12.4%)		
AIH	24 (18.9%)	7 (31.8%)	17 (16.2%)		
Other	32 (25.2%)	6 (27.3%)	26 (24.7%)		
Blood pressure					
SBP (mm Hg)	119.30 ± 15.05	118.55 ± 19.26	119.46 ± 14.13	0.257	0.797
DBP (mm Hg)	73.21 ± 11.61	72.09 ± 13.25	73.45 ± 11.29	0.448	0.658
Ammonia (μmoL/L)	32.00 ± 22.60	31.29 ± 24.22	32.15 ± 26.06	0.149	0.882
Albumin (g/L)	33.09 ± 4.91	32.17 ± 4.73	33.28 ± 4.95	0.991	0.329
TBil(μmoL/L)	29.63 ± 26.09	34.26 ± 25.30	28.67 ± 26.26	−0.913	0.363
Cr(μmoL/L)	81.77 ± 118.02	66.88 ± 21.34	84.89 ± 129.34	0.649	0.517
**Child-pugh class**				29.272	**< 0.001**
A	55 (43.3%)	2 (9.1%)	53 (50.4%)		
B	64 (50.4%)	13 (59.1%)	51 (48.6%)		
C	8 (6.3%)	7 (31.8%)	1 (1.0%)		
MELD score (median, IQR)	7.78 ± 5.03	7.82 ± 3.52	7.77 ± 5.31	−0.042	0.967
**FBG (mmol/L)**	5.82 ± 2.27	8.17 ± 4.36	5.33 ± 0.98	−6.068	**< 0.001**
TC (mmoL/L)	2.86 ± 0.88	2.72 ± 0.88	2.89 ± 0.88	0.839	0.403
TG (mmoL/L)	0.90 ± 0.55	0.94 ± 0.42	0.89 ± 0.57	−0.312	0.756
**HDL-C (mmol/L)**	0.93 ± 0.29	0.80 ± 0.25	0.96 ± 0.29	2.445	**0.016**
LDL-C (mmol/L)	1.74 ± 0.58	1.74 ± 0.55	1.74 ± 0.59	−0.037	0.971
**Diabetes**	34 (26.8%)	11 (50.0%)	23 (21.9%)	7.324	**0.010**
Hyperlipidemia	1 (0.8%)	1 (4.5%)	0 (0%)	4.773	0.173
**Hypertension**	18 (14.2%)	8 (36.4%)	10 (9.5%)	8.772	**0.003**
**MetS**	24 (18.9%)	9 (40.9%)	15 (14.3%)	7.231	**0.007**

Values are the mean ± SD or n (%). MHE, minimal hepatic encephalopathy; PHES, psychometric hepatic encephalopathy score; BMI, body mass index; AIH, autoimmune hepatis; SBP, systolic blood pressure; DBP, diastolic blood pressure; TBil, total bilirubin; MELD, model for end-stage liver disease; FBG, fasting blood glucose; TC, total cholesterol; TG, triglyceride; HDL-C, high density lipoprotein cholesterol; LDL-C, low density lipoprotein cholesterol; MetS, metabolic syndrome. Bold values indicate statistical significance (*P* < 0.05).

### 3.2 Relationship between MetS and its components with MHE

Multivariate logistics regression (Table 2) showed that FBG is an independent risk factors for MHE in patients with portal hypertension after adjusting for age, gender, Child-Pugh Class [OR = 1.743, 95%CI (1.173–2.590), *P* = 0.006]. In a separate model replacing FBG with diabetes, diabetes was also independently associated with MHE (OR = 5.194, 95% CI: 1.461–18.461, *P* = 0.011). To validate the association, modified Poisson regression analysis was used to evaluate the relationship (Table 3). FBG [β = 2.473, 95%CI (1.051–5.818), *P* = 0.038] and diabetes [β = 2.225, 95%CI (1.160–4.268), *P* = 0.016] were significantly associated with the incidence of MHE, which were consistent with the logistic results, suggesting that FBG and diabetes were risk factors for MHE.

**TABLE 2:** Logistic regression analysis of the risk of minimal hepatic encephalopathy associated with metabolic syndrome in patients with portal hypertension.

Univariate analysis			Multivariate analysis	
	OR (95%CI)	*P*	OR (95%CI)	*P*
SBP	0.996 (0.965–1.027)	0.795		
DBP	0.990 (0.951–1.030)	0.617		
BMI	0.952 (0.842–1.077)	0.434		
**FBG**	**1.921 (1.329**–**2.775)**	**< 0.001**	**1.743 (1.173**–**2.590)**	**0.006**
**Diabetes**	**3.565 (1.372**–**9.266)**	**0.009**		
TG	1.135 (0.514–2.509)	0.754		
HDL-C	0.090 (0.012–0.656)	0.090		
Hyperlipidemia	3.191 (–0.275–7.686)	0.074		
**Hypertension**	**5.429 (1.833**–**16.080)**	**0.002**		
**MetS**	**4.154 (1.512**–**11.410)**	**0.006**		

Logistic regression analysis adjusting for age, gender, and Child-pugh class. Diabetes was analyzed in a separate model (replacing FBG) and remained independently associated with MHE (OR = 5.194, 95% CI: 1.461–18.461, *P* = 0.011). FBG, fasting blood glucose; HDL-C, high density lipoprotein cholesterol; MetS, metabolic syndrome; OR, odds ratio; CI, confidence interval. Bold values indicate statistical significance (*P* < 0.05).

**TABLE 3:** Modified Poisson regression analysis of the risk of minimal hepatic encephalopathy in patients with cirrhosis and portal hypertension.

Character	β	aRR (95%CI)	Wald χ ^2^	*P*
SBP	1.001	0.972–1.031	0.008	0.928
DBP	1.009	0.983–1.037	0.485	0.486
BMI	0.973	0.891–1.063	0.363	0.547
**FBG**	**2.473**	**1.051**–**5.818**	**4.304**	**0.038**
**Diabetes**	**2.225**	**1.160**–**4.268**	**5.795**	**0.016**
TG	0.930	0.540–1.603	0.068	0.795
HDL-C	0.317	0.078–1.290	2.576	0.109
**Hyperlipidemia**	**2.092**	**1.106**–**3.957**	**5.154**	**0.023**
Hypertension	1.612	0.929–2.798	2.882	0.090
**MetS**	**2.089**	**1.178**–**3.702**	**6.357**	**0.012**

Modified Poisson regression analysis adjusting for age, gender, and Child-pugh class. FBG, fasting blood glucose; aRR, adjusted relative risk; CI, confidence intervals. Bold values indicate statistical significance (*P* < 0.05).

Table 4 shows the results of multiple linear regression analysis for each component of MetS and PHES scores. Among the factors, FBG (β = −0.289, 95%CI (−0.520, −0.057), *P* = 0.015) was negatively associated with PHES score. However, Other components of MetS such as SBP, DBP, BMI, Diabetes, TG, HDL-C, etc. showed that their effects on PHES scores were not statistically significant (*P* > 0.05). Overall, higher FBG was risk factor that had a significant impact on MHE.

**TABLE 4:** Multiple linear regression was used to investigate the association between metabolic syndrome components and psychometric hepatic encephalopathy scores.

Characteristics	β	95%CI	*t*	*P*
SBP	0.020	–0.013 to –0.053	1.176	0.242
DBP	0.009	–0.034 to –0.052	0.402	0.688
BMI	0.057	–0.078 to –0.191	0.835	0.406
**FBG**	–**0.289**	–**0.520 to** –**0.057**	–2**.469**	**0.015**
Diabetes	–0.578	–1.743 to –0.586	–0.983	0.328
TG	0.441	–0.490 to –1.371	0.938	0.350
HDL-C	0.786	–0.935 to –2.508	0.904	0.368
Hyperlipidemia	–3.503	–9.156 to –2.150	–1.227	0.222
Hypertension	–0.284	–1.882 to –1.115	–0.507	0.613
MetS	–0.457	–1.794 to –0.879	–0.678	0.499

Multiple linear regression adjusting for age, gender, and Child-pugh class. BMI, body mass index; SBP, systolic blood pressure; DBP, diastolic blood pressure; FBG, fasting blood glucose; TC, total cholesterol; TG, triglyceride; HDL-C, high density lipoprotein cholesterol; LDL-C, low density lipoprotein cholesterol; CI, confidence intervals. Bold values indicate statistical significance (*P* < 0.05).

### 3.3 Dose-response relationship between MetS components and MHE

**FIGURE 1:**
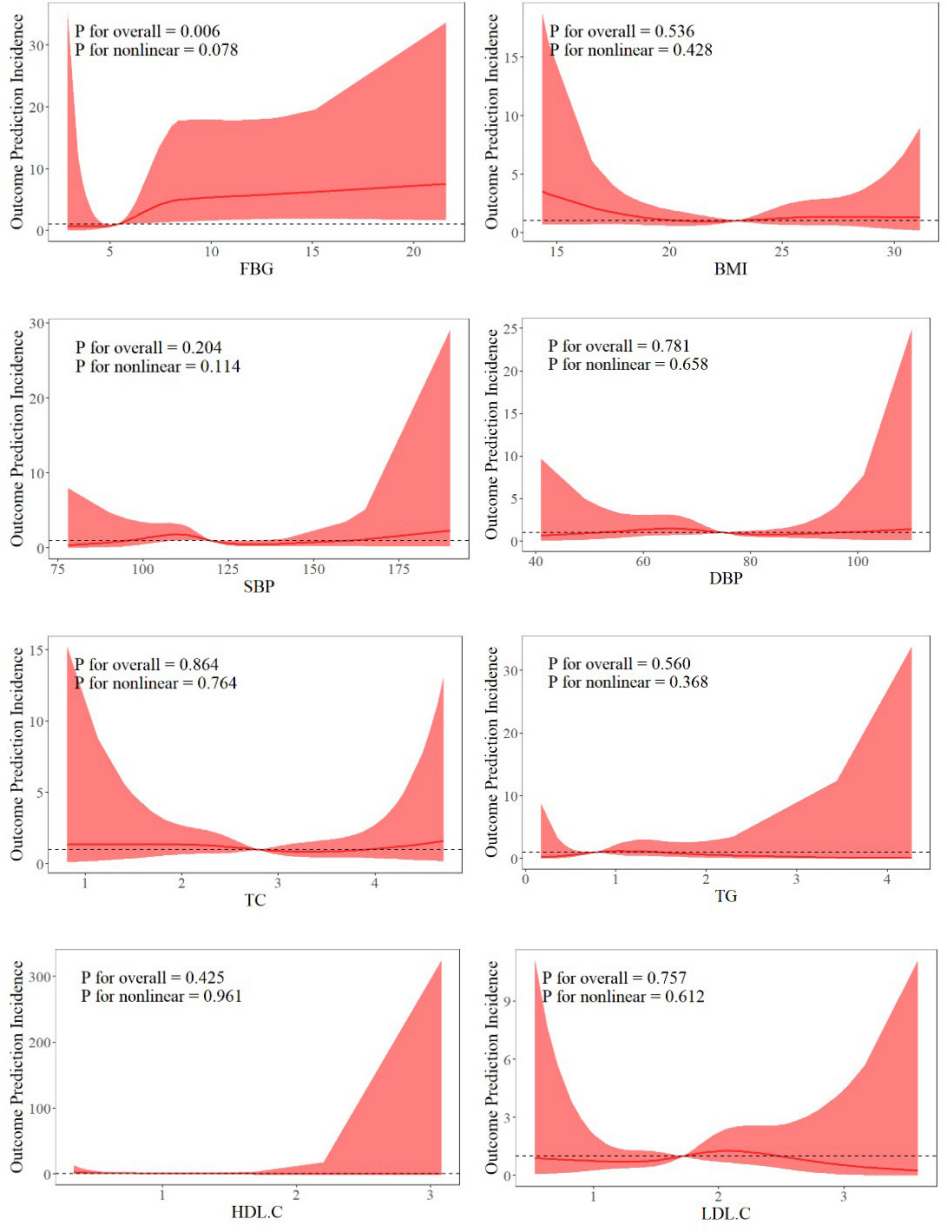
Restricted cubic spline analysis of the correlation between metabolic syndrome and minimal hepatic encephalopathy.

Figure 1 illustrates the nonlinear association between components of MetS and MHE in patients with cirrhosis and portal hypertension. The association was constructed using a smoothed curve of a generalized additive logistic model. The inflection point of FBG was determined to be 8.36 by two-stage logistic regression analysis (Supplementary Table S1). The results showed that OR was 1.921 (95% confidence interval: 1.274–2.896, *P* < 0.05) when FBG < 8.36. These findings suggest that below the inflection point value, higher FBG levels were associated with a significantly increased risk of MHE in patients with cirrhosis and portal hypertension. 94.5% of all patients in this study had an FBG level below 8.36 mmoL/L, indicating that the statistically significant association between elevated FBG and increased MHE risk was applicable to the vast majority of the study population, further supporting the clinical value of blood glucose management in this cohort.

### 3.4 Relationship between MetS components and PHES subscale performance

**FIGURE 2:**
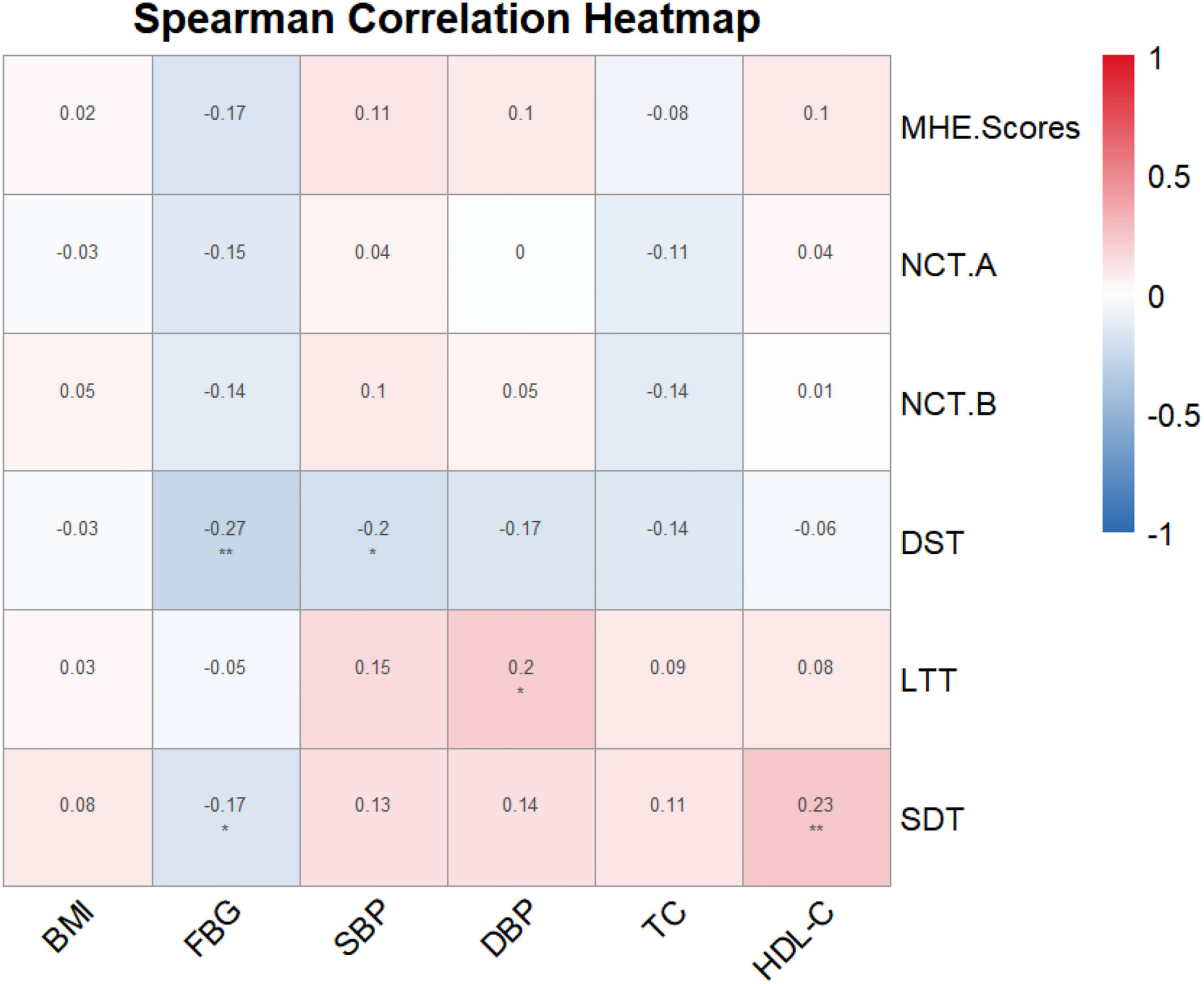
Spearman correlation heatmap of metabolic parameters and PHES subscale scores. *P < 0.05, **P < 0.01.

Spearman correlation analysis (Figure 2) revealed that FBG was significantly negatively correlated with DST (*r* = -0.27, *P* < 0.01) and SDT (*r* = -0.17, *P* < 0.05) scores, indicating that higher glucose levels were associated with poorer psychomotor speed, attention, and visual search performance. SBP was also negatively correlated with DST scores (*r* = -0.20, *P* < 0.05), while DBP was positively correlated with LTT scores (*r* = 0.20, *P* < 0.05). No significant correlations were found between other metabolic parameters and PHES subscale scores (all *P* > 0.05).

### 3.5 Risk factors of MHE

Univariate and multivariable logistic regression analysis showed that age (OR = 1.103, 95% CI 1.027–1.184, *P* = 0.007), Child-Pugh Class (OR = 5.869, 95% CI 1.588–21.688, *P* = 0.008), FBG (OR = 1.750, 95% CI 1.164–2.630, *P* = 0.007) were independent predictors of MHE (Table 5).

**TABLE 5:** Logistic regression analysis of the risk of minimal hepatic encephalopathy in patients with cirrhosis and portal hypertension.

Univariate analysis			Multivariate analysis	
	OR (95%CI)	*P*	OR (95%CI)	*P*
Age	1.128 (1.063–1.198)	< 0.001	1.103 (1.027–1.184)	0.007
Gender	4.107 (1.536–10.982)	0.005		
AIH	4.035 (1.129–14.417)	0.032		
Child-pugh	12.384 (3.668–41.814)	< 0.001	5.869 (1.588–21.688)	0.008
FBG	1.921 (1.329–2.775)	< 0.001	1.750 (1.164–2.630)	0.007
HDL-C	0.090 (0.012–0.656)	0.017		
Hypertension	5.429 (1.833–16.080)	0.002		
Diabetes	3.565 (1.372–9.266)	0.009		
MetS	4.154 (1.512–11.410)	0.006		

Diabetes was evaluated in a separate model and showed a similar independent association with MHE (OR = 4.702, 95% CI: 1.368–16.158, *P* = 0.014). AIH, autoimmune hepatis; FBG, fasting blood glucose; HDL-C, high density lipoprotein cholesterol; MetS, metabolic syndrome; CI, confidence intervals; OR, odds ratio.

## 4 Discussion

In recent years, MetS has become a significant global public health concern, afflicting adults in an increasing number of developed and developing countries (Prasun, 2020). Our study identified an exploratory nonlinear threshold effect of FBG on MHE in patients with MetS, suggesting a potential target for blood glucose management in cirrhotic patients (<8.36 mmoL/L) that warrants further validation. Through multi-model validation, it was confirmed that FBG and diabetes were interventionable risk factors independent of the severity of liver disease, and their damage patterns to specific cognitive domains were clarified. Clinically, our findings advocate for integrating MetS screening into routine HE risk assessment.

Existing reports have already revealed the relationship between diabetes and hepatic encephalopathy following cirrhosis (Gairing et al., 2023; Jepsen et al., 2015; Liu et al., 2016). This study also confirmed the effect of diabetes mellitus on the minimal occurrence of hepatic encephalopathy in patients with cirrhosis. Of the 22 patients with hepatic encephalopathy in the decompensated stage of cirrhosis, 11 (50%) had diabetes. Our study demonstrates that metabolic dysregulation, particularly hyperglycemia and diabetes mellitus, serves as an independent predictor of MHE in cirrhotic patients with portal hypertension. The liver is the central organ of glucose homeostasis, responsible for glycogen synthesis, gluconeogenesis, and insulin clearance. In cirrhosis, hepatocellular injury and portal hypertension severely disrupt hepatic glucose regulation, leading to insulin resistance and hyperglycemia. When diabetes coexists, the liver’s ability to maintain glucose stability is further compromised, which in turn aggravates brain insulin resistance, oxidative stress, and ammonia metabolism disorders, ultimately promoting the development of MHE. This study strongly supports the new clinical concept of diabetes-hepatic joint management, which advocates that liver function and blood glucose should be jointly evaluated and managed in patients with cirrhosis (Castera and Cusi, 2023; Chronic Disease Management Branch and China Pharmaceutical Biotechnology Association, 2022; Mohan et al., 2025). Blood glucose control should be incorporated into routine clinical management of cirrhotic patients to reduce the risk of MHE. Notably, diabetes and hyperglycemia are risk factors, not determinants, of MHE. Not all patients with uncontrolled diabetes develop MHE. In contrast, overt hepatic encephalopathy is more closely associated with renal failure and other acute complications. Our study focused on early minimal hepatic encephalopathy, in which glucose dysregulation plays a distinct and independent role.

FBG exhibited a nonlinear relationship with MHE risk, characterized by an inflection point at 8.36 mmoL/L. Below this threshold, each 1 mmoL/L increase in FBG was associated with a 92.1% elevated risk of MHE (OR = 1.921, *P* < 0.05), whereas no significant association was observed above 8.36 mmoL/L. This finding aligns with prior studies linking diabetes to overt HE, yet extends current knowledge by suggesting a potential glycemic threshold for MHE risk stratification that requires further validation (Ampuero et al., 2013; Gairing et al., 2023). The identified FBG inflection point (8.36 mmol/L) may reflect a pathophysiological transition in glucose metabolism, although this finding is exploratory and requires confirmation (Wei and Zhang, 2024). Below this threshold, moderate hyperglycemia likely exacerbates hepatic insulin resistance and promotes ammonia generation through glutaminase activation in skeletal muscle and gut microbiota (Petrides and DeFronzo, 1989; Petrides et al., 1994; Rizo-Roca et al., 2025). Concurrently, impaired hepatic urea cycle function in cirrhosis further amplifies systemic ammonia load. However, when FBG exceeds 8.36 mmol/L, the lack of association with MHE risk might be tentatively attributed to competing pathways, such as intensive glycemic control masking the true metabolic burden or severe hyperglycemia precipitating acute complications that independently worsen encephalopathy (Jepsen et al., 2015; Liu et al., 2016). If confirmed in future studies, this exploratory nonlinearity could inform tailored glycemic targets in cirrhotic populations. To our knowledge, relatively few studies have examined the nonlinear relationship between FBG and MHE risk. Our exploratory analysis suggests a potential threshold effect that requires confirmation in independent cohorts.

The PHES scale measures the patient’s attention, psychomotor speed and accuracy, and visuospatial perception (Weissenborn, 2008). The NCT-A and NCT-B mainly test the subject’s attention and the brain’s processing and executive function of simple number sequences; In addition to cognitive dysfunction, hepatic encephalopathy may also lead to a decrease in motor speed or motor accuracy, and LTT can judge the patient’s psychomotor speed and accuracy; DST is mainly used to assess the brain’s visual-motor integration function, learning ability, memory, and executive function; SDT mainly tests the subject’s visual search ability, motor speed, accuracy, and attention sharing (Coubard et al., 2021; Kircheis et al., 2014; Weissenborn, 2008). Our data demonstrated a dose-dependent inverse correlation between FBG levels and performance on NCT-A, DST, and SDT subtests. This suggests that hyperglycemia-driven oxidative stress and cerebral insulin resistance may preferentially target the prefrontal-hippocampal circuitry, disrupting attentional allocation and visuomotor integration (Barone et al., 2021; Masenga et al., 2023; Ryuk et al., 2022).

There are several limitations to consider. First, retrospective design excludes causal inference, and unmeasured confounders may bias the results. Second, this is a single-center study with a likely small sample size, which may limit the generalizability of our findings and the precision of some effect estimates. Larger prospective cohorts are needed to validate our results. But the test power of our study was sufficient. What is more, some key metabolic indicators for evaluating metabolic syndrome, such as insulin, hs-CRP, and uric acid, were not routinely measured and could not be included. In addition, our research has been verified by multiple models to ensure the authenticity and generalizability of the research results.

## Data Availability

The original contributions presented in the study are included in the article/Supplementary material, further inquiries can be directed to the corresponding authors.
