# Supplementary material for: Associations of metabolic syndrome with minimal hepatic encephalopathy in patients with cirrhosis and portal hypertension: a retrospective cohort study
Source: Front Behav Neurosci. 2026 May 20;20:1779609. doi: 10.3389/fnbeh.2026.1779609 (PMC13230085; doi:10.3389/fnbeh.2026.1779609)
Supplement: Supplementary file 1 [file data_sheet_1.docx]

Table S1. A two-stage logistic regression model was used to perform threshold analysis on the impact of each component of metabolic syndrome on minimal hepatic encephalopathy.

| Outcome | Adjusted 95%CI | P value |
| --- | --- | --- |
| FBG |  |  |
| Fitting model by standard linear regression | 1.133(1.047-1.226) | 0.002 |
| Fitting model by two-piecewise linear regression |  |  |
| Inflection point | 8.36 |  |
| FBG<8.36 | 1.921(1.274-2.896) | 0.002 |
| FBG>8.36 | 0.983(0.850-1.137) | 0.817 |
| P for likelihood ratio test | 0.011 |  |
| BMI |  |  |
| Fitting model by standard linear regression | 0.972(0.868-1.089) | 0.629 |
| Fitting model by two-piecewise linear regression |  |  |
| Inflection point | 21.51 |  |
| BMI<21.51 | 0.824(0.650-1.045) | 0.111 |
| BMI>21.51 | 1.070(0.905-1.266) | 0.427 |
| P for likelihood ratio test | 0.159 |  |
| SBP |  |  |
| Fitting model by standard linear regression | 0.991 (0.964-1.008) | 0.507 |
| Fitting model by two-piecewise linear regression |  |  |
| Inflection point | 136 |  |
| SBP<136 | 0.977(0.947-1.008) | 0.138 |
| SBP>136 | 1.029(0.982-1.078) | 0.232 |
| P for likelihood ratio test | 0.156 |  |
| DBP |  |  |
| Fitting model by standard linear regression | 0.991(0.956-1.027) | 0.612 |
| Fitting model by two-piecewise linear regression |  |  |
| Inflection point | 90 |  |
| DBP<90 | 0.979(0.942-1.018) | 0.293 |
| DBP>90 | 1.081(0.952-1.227) | 0.229 |
| P for likelihood ratio test | 0.243 |  |
| TC |  |  |
| Fitting model by standard linear regression | 0.869(0.543-1.391) | 0.559 |
| Fitting model by two-piecewise linear regression |  |  |
| Inflection point | 4.24 |  |
| TC<4.24 | 0.983(0.583-1.659) | 0.949 |
| TC>4.24 | 0 (0-Inf) | 0.997 |
| P for likelihood ratio test | 0.177 |  |
| TG |  |  |
| Fitting model by standard linear regression | 0.951(0.488-1.855) | 0.883 |
| Fitting model by two-piecewise linear regression |  |  |
| Inflection point | 0.89 |  |
| TG<0.89 | 20.478(0.815-514.756) | 0.066 |
| TG>0.89 | 0.412(0.093-1.831) | 0.244 |
| P for likelihood ratio test | 0.038 |  |
| HDL-C |  |  |
| Fitting model by standard linear regression | 0.282(0.060-1.338) | 0.111 |
| Fitting model by two-piecewise linear regression |  |  |
| Inflection point | 0.54 |  |
| HDL-C<0.54 | 0.001(0-1.808) | 0.069 |
| HDL-C>0.54 | 0.496(0.1-2.467) | 0.392 |
| P for likelihood ratio test | 0.169 |  |
| LDL-C |  |  |
| Fitting model by standard linear regression | 1.141(0.561-2.322) | 0.715 |
| Fitting model by two-piecewise linear regression |  |  |
| Inflection point | 2.6 |  |
| LDL-C<2.6 | 1.881 (0.745-4.747) | 0.181 |
| LDL-C>2.6 | 0 (0-44191.221) | 0.292 |
| P for likelihood ratio test | 0.059 |  |

FBG, fasting blood glucose; BMI, body mass index; SBP, systolic blood pressure; DBP, diastolic blood pressure; TC, total cholesterol; TG, triglyceride; HDL-C, high density lipoprotein cholesterol; LDL-C, low density lipoprotein cholesterol; CI, confidence intervals.

Table S2. Sample size calculation and post-hoc power analysis

| **Item** | **Value** |
| --- | --- |
| MHE / No MHE | 22 / 105 |
| FBG (mmol/L), mean±SD | 8.17±4.36 / 5.33±0.98 |
| Mean difference | 2.84 |
| α (two-sided) | 0.05 |
| Target power | 80% |
| Minimum required sample size | 102 |
| Actual sample size | 127 |
| Achieved power | 84.30% |
| Events per variable (EPV) | 5.5 |
| Conclusion | Adequate |
